# Age-Related DNA Methylation Changes: Potential Impact on Skeletal Muscle Aging in Humans

**DOI:** 10.3389/fphys.2019.00996

**Published:** 2019-08-02

**Authors:** Noémie Gensous, Maria Giulia Bacalini, Claudio Franceschi, Carel G. M. Meskers, Andrea B. Maier, Paolo Garagnani

**Affiliations:** ^1^Department of Experimental, Diagnostic and Specialty Medicine, University of Bologna, Bologna, Italy; ^2^IRCCS Istituto delle Scienze Neurologiche di Bologna, Bologna, Italy; ^3^Lobachevsky State University of Nizhny Novgorod, Nizhny Novgorod, Russia; ^4^Amsterdam UMC, Department of Rehabilitation Medicine, Amsterdam Movement Sciences, Vrije Universiteit Amsterdam, Amsterdam, Netherlands; ^5^Department of Human Movement Sciences, @AgeAmsterdam, Faculty of Behavioural and Movement Sciences, Amsterdam Movement Sciences, Vrije Universiteit Amsterdam, Amsterdam, Netherlands; ^6^Department of Medicine and Aged Care, @AgeMelbourne, The Royal Melbourne Hospital, The University of Melbourne, Melbourne, VIC, Australia; ^7^Clinical Chemistry, Department of Laboratory Medicine, Karolinska Institutet, Karolinska University Hospital, Stockholm, Sweden; ^8^Applied Biomedical Research Center (CRBA), Policlinico S.Orsola-Malpighi Polyclinic, Bologna, Italy; ^9^CNR Institute of Molecular Genetics, Unit of Bologna, Bologna, Italy

**Keywords:** aging, muscle, sarcopenia, epigenetics, DNA methylation, epigenetic clocks

## Abstract

Human aging is accompanied by a decline in muscle mass and muscle function, which is commonly referred to as sarcopenia. Sarcopenia is associated with detrimental clinical outcomes, such as a reduced quality of life, frailty, an increased risk of falls, fractures, hospitalization, and mortality. The exact underlying mechanisms of sarcopenia are poorly delineated and the molecular mechanisms driving the development and progression of this disorder remain to be uncovered. Previous studies have described age-related differences in gene expression, with one study identifying an age-specific expression signature of sarcopenia, but little is known about the influence of epigenetics, and specially of DNA methylation, in its pathogenesis. In this review, we will focus on the available knowledge in literature on the characterization of DNA methylation profiles during skeletal muscle aging and the possible impact of physical activity and nutrition. We will consider the possible use of the recently developed DNA methylation-based biomarkers of aging called epigenetic clocks in the assessment of physical performance in older individuals. Finally, we will discuss limitations and future directions of this field.

## Introduction

Human aging is accompanied with a decline in skeletal muscle (SM) mass and function, which is commonly referred to as sarcopenia. It negatively affects the quality of life of older people as it is associated with decreased mobility, loss of physical independence and increased morbidity and mortality (Ling et al., 2010; Szulc et al., 2010; Taekema et al., 2010; Beaudart et al., 2017). Sarcopenia represents a major public health problem, that is anticipated to grow in the next decades due to the increase in life expectancy (Ethgen et al., 2017; Shafiee et al., 2017). Sarcopenia is a complex multifactorial phenomenon, encompassing both intrinsic (endocrine factors, motor neuron loss, mitochondrial dysfunction) and extrinsic factors (nutrition, exercise) (Cruz-Jentoft et al., 2010; Kim and Choi, 2013), but the precise underlying molecular mechanisms remain poorly characterized. Regulation of gene expression is a fundamental factor that impacts the phenotype of each tissue and its age-related alterations are involved in the aging process (Roy et al., 2002). Previously published studies have tried to decipher the transcriptomic profiles associated with SM aging, and age-related differences in gene expression have been described (Jozsi et al., 2000; Roth et al., 2002; Welle et al., 2003, 2004; Zahn et al., 2006; Melov et al., 2007; Raue et al., 2012; Liu et al., 2013; Mamoshina et al., 2018; Shafiee et al., 2018). Genes that exhibit different expressions in relation to age are, among other, involved in metabolism, stress responses, control of the cell cycle and apoptosis, DNA damage response and transcriptional regulation. Giresi et al. (2005) were able to identify an age-specific expression signature of sarcopenia, comprised of 45 genes that best distinguished the vastus lateralis muscle of young from old male subjects. If the transcriptomic profiles associated with muscle aging constitute a first essential level of information, the characterization of other layers of genomic regulation (both pre- and post-transcriptional) could be informative and allow to better understand the process of muscle aging. Among them, epigenetic mechanisms, which refer to changes in gene function that are not related to changes in the DNA sequence itself, are subject to profound rearrangements during aging. Current data clearly demonstrate that the rearrangements in the epigenetic landscape are one major hallmark of the aging process (López-Otín et al., 2013; Kennedy et al., 2014). DNA methylation, which corresponds to the covalent addition of a methyl group to the cytosine in a CpG dinucleotide, is one of the best studied and most mechanistically understood epigenetic mechanism. Its role in aging, its implication in cellular senescence and in the development of various diseases has been extensively investigated (Calvanese et al., 2009; Pal and Tyler, 2016; Gensous et al., 2017). However, to date, our understanding of its role in muscle aging is far from complete. The aim of this article is to review the available evidence in humans on the epigenetics of muscle aging, focusing specially on DNA methylation. Additionally, we will also discuss the potential impact of physical activity and nutrition in these processes as well as the possible use of the newly developed epigenetic biomarkers of aging (also referred to as epigenetic clocks) in the assessment of physical performance and sarcopenia in the older individuals. We will finally specifically discuss the limitations and future directions of this field.

## Literature Search

For this narrative review, the electronic search involved three databases (PubMed, Scopus, and Google Scholar) and included the following search terms: (epigenetics OR “DNA methylation” OR “epigenetic clock”) AND (sarcopenia OR “muscle weakness” OR “muscular weakness” OR “muscular atrophy”) AND (“physical activity” OR nutrition). The inclusion criteria were original research articles, articles published in English language and related to humans. Non-human studies were scarce and were not included. Reviews were excluded. Reference lists of selected papers were hand searched for additional relevant publications.

## Epigenetics of Human Skeletal Muscle Aging: Focus on DNA Methylation

Aging is characterized by a marked remodeling of genomic DNA methylation patterns, with four main different types of changes (Pal and Tyler, 2016): global hypomethylation, differential methylation of specific genomic loci, increase in inter-individual divergence between patterns of DNA methylation and increase in the rate of epimutations. During the last decades, extensive work has been carried out to relate the epigenetic changes to aging and age-related phenotypes, and this remains an active area of research. Various methods are available for determining the methylation status of DNA samples (Kurdyukov and Bullock, 2016) and in the following paragraphs, we will review data regarding human muscle aging obtained with candidate gene approaches or with genome-wide analysis. We will also discuss the potential impact of DNA methylation age-related changes on satellite cells.

### Gene-Specific DNA Methylation Changes Associated With Muscle Aging

In the late 2000s, two studies examined methylation levels of candidate genes in the vastus lateralis SM from young versus old healthy subjects (Ling et al., 2007; Rönn et al., 2008; Table 1). Methylation patterns of two genes coding for components of the respiratory chain, *NDUFB6* (NADH:Ubiquinone Oxidoreductase Subunit B6) and *COX7A1* (Cytochrome C Oxidase Subunit 7A1), were analyzed. In both cases an age-related increase in DNA methylation, combined with a decrease in gene expression, was observed (Ling et al., 2007; Rönn et al., 2008), suggesting the influence of DNA methylation on the expression of metabolically important genes in SM and its possible implication in the susceptibility to age-related metabolic diseases.

**TABLE 1:** DNA methylation profiles associated with muscle aging.

Author	Year	Number of subjects	Age (years)	Females, *n* (%)	Location	Population	Measure of physical functioning	Tissue for DNA methylation analysis	DNA methylation analysis	Main result
**Candidate gene analysis**										
Ling et al.	2007	196	*Group of young subjects*: mean = 28.0 (SD = 1.9) *Group of old subjects*: mean = 62.4 (SD = 2.0)	*Young group*: 50 (45.4%) *Old group*: 48 (55.8%)	Denmark/Sweden	Healthy twin subjects	NA	Skeletal muscle	Bisulfite sequencing	Increased DNA methylation in the promoter of the *NDUFB6* gene in elderly subjects compared to young ones
Rönn et al.	2008	196	*Group of young subjects*: mean = 28.0 (SD = 1.9) *Group of old subjects*: mean = 62.4 (SD = 2.0)	*Young group*: 50 (45.4%) *Old group*: 48 (55.8%)	Denmark/Sweden	Healthy twin subjects	NA	Skeletal muscle	Bisulfite sequencing	Increased DNA methylation in the promoter of the *COX7A1* gene in elderly subjects compared to young ones
**Genome-wide analysis**										
Zykovich et al.	2014	48	*Group of young subjects:* mean = 21.3 (SD = 2.4) *Group of old subjects*: mean = 73.2 (SD = 4.6)	0 (0%)	Canada – United States	Healthy subjects	NA	Skeletal muscle	Illumina Human Methylation 450K	Predominant pattern of DNA hypermethylation in the aged group. 5963 individual CpG sites differentially methylated between the two groups, with 500 of them used to develop an epigenetic signature of muscle aging
Livshits et al.	2016	1550	Mean = 51.8 (SD = 13.17)	1550 (100%)	United Kingdom	Healthy twin subjects	Whole body dual-energy X-ray absorptiometry method	Whole blood	Methylated DNA immuno-precipitation sequencing	Identification of seven regions whose methylation status was significantly associated with variation in skeletal muscle mass
**EWAS with markers of physical fitness**										
Bell et al.	2012	172	Median = 57 (range = 32–80)	172 (100%)	United Kingdom	Healthy subjects	HGS	Whole blood	Illumina Human Methylation 27K	No differentially methylated region associated with HGS
Marioni et al.	2015b	1091	Mean = 69.5 (SD = 0.83)	543 (49.8%)	United Kingdom	Healthy subjects	6 m walking test and HGS	Whole blood	Illumina Human Methylation 450K	No significant association between individual CpG methylation sites and HGS or 6 m walking speed

*SD, standard deviation; HGS, hand grip strength; NA, not applicable.*

### Genome-Wide DNA Methylation Analysis

Development of large-scale technologies has greatly changed the study of epigenomics in the last years and has led to significant advances in the understanding of DNA methylation changes during aging. Some studies have applied genome-wide technologies to the study of muscle aging, providing a comprehensive profiling of DNA methylation patterns in this process (Table 1). The first genome-wide study of DNA methylation dynamics in SM was published by Zykovich et al. (2014). Samples collected from 24 healthy older male adults (age range: 68–89 years) were compared to 24 younger ones (age range: 18–27 years). A predominant pattern of DNA hypermethylation throughout the genome was observed within the aged group. The authors identified specifically 5963 individual CpG sites that were differentially methylated between the two groups. Of these, 5518 (92%) were hypermethylated with age, while the remaining were hypomethylated. Surprisingly, hypermethylation of CpG dinucleotides with age occurred mainly within gene bodies (middle and 3′ end of genes), rather than in the promoter regions, and differentially methylated positions (DMPs) were underrepresented outside of genes. When the authors performed an ontology analysis on the intragenic methylation changes, they observed that the most enriched terms and pathways were related to “muscle cell” and “axon guidance signaling,” suggesting a potential role of epigenetic changes in the denervation of the neuromuscular junction during aging (Gonzalez-Freire et al., 2014). Finally, among the 5963 DMPs, Zykovich et al. (2014) selected 500 CpG sites which were able to discriminate with high confidence young tissues from older ones, thus defining the first epigenetic signature of muscle aging. To our knowledge, this is the only epigenome-wide study conducted in human SM samples of healthy individuals during aging. Another one, published 2 years later, aimed to also identify associations between DNA methylation levels at some CpG sites and SM mass variation with age, but it was performed on whole blood (Livshits et al., 2016). In a population of 1550 middle-aged female twins (age range: 17–82 years), authors identified seven regions whose methylation status was significantly associated with variation in SM mass. Four of these CpG sites of interest were located in or near the genes *DNAH12* (Dynein Axonemal Heavy Chain 12), *CAND1* (Cullin Associated And Neddylation Dissociated 1), *CYP4F29P* (Cytochrome P450 Family 4 Subfamily F Member 29, Pseudogene), and *ZFP64* (ZFP64 Zinc Finger Protein), previously identified for some of them (*DNAH12, ZFP64)* as involved in muscle physiology (Teran-Garcia et al., 2005; Sakamoto et al., 2008). Finally, two observational epidemiological studies with epigenome-wide association analysis (EWAS) have tested associations between whole-blood DNA methylation patterns and markers of physical fitness (Bell et al., 2012; Marioni et al., 2015b; Table 1). In a cohort of 172 female twins aged from 32 to 80 years old, no association between methylation levels at individual CpG sites and hand grip strength (HGS) was found (Bell et al., 2012). Similar results were observed in another cohort of 1091 individuals: there were no significant association between individual CpG methylation sites and HGS or 6 m walking speed (Marioni et al., 2015b).

### Satellite Cells

Additional research is required to determine the contribution of DNA methylation to muscle aging and to the development of sarcopenia and one promising line of research could be the investigation of the impact of age-related epigenetic changes on SM stem cell population, known as satellite cells. These cells, located between the basal lamina and the muscle fiber sarcolemma, contribute to muscle tissue turnover, repair and regeneration (Dumont et al., 2015). Aging is accompanied by reduced satellite cell pools and by a global decrease in their functional properties (Renault et al., 2002; Dumont et al., 2015). This is believed to exacerbate the decline of muscle mass and strength associated with sarcopenia (Renault et al., 2002). Epigenetic factors, and specially DNA methylation, are involved in satellite cells differentiation and activation during early life, essentially by their capacity to modify gene expression profiles (Berdasco and Esteller, 2011; Dilworth and Blais, 2011). DNA methylation could also contribute to the decline of myogenic capacity of satellite cells during aging. Of particular interest are the data obtained by Bigot et al. (2015). To our knowledge, this is the only study performed on human cells *in vitro*, specifically dedicated to the SM aging process. In this study, a general age-related hypermethylation of gene bodies in elderly muscle stem cells was observed as compared to cells isolated from young subjects, echoing the data obtained in post-mitotic skeletal muscle (Zykovich et al., 2014). It was also demonstrated an impaired capacity from stem cell self-renewal in elderly muscle (Bigot et al., 2015). This impaired self-renewal was linked to an increased methylation with age of the *SPRY1* (Sprouty RTK Signaling Antagonist 1) gene, previously described as a regulator of muscle stem cell quiescence (Shea et al., 2010). The increased methylation of *SPRY1*, associated with a reduction in its transcription, could be responsible of a failure of re-quiescence in activated stem cells, leading to a decline of their pool in elderly human muscle (Bigot et al., 2015).

## Impact of Physical Activity and Nutrition on DNA Methylation Profiles of Skeletal Muscle Aging

DNA methylation patterns are not fixed but dynamic, and they can be deeply modulated by environmental factors. They represent a fundamental molecular link between aging and environment. Physical activity and diet have been investigated for their potential influence on DNA methylation profiles in several tissues (Rönn et al., 2013; Bacalini et al., 2014). In humans, regular physical activity and healthy eating have been associated with major health benefits, including a global reduction in morbidity and mortality (Warburton et al., 2006; Kopperstad et al., 2017). There is also now growing evidence indicating that resistance training can improve muscle mass, muscle strength, functional mobility, and balance in older adults (Papa et al., 2017). The molecular mechanisms behind the beneficial effects of physical activity and healthy diet on SM function are not fully understood, but we can assume that one of the mechanisms through which they may induce their beneficial effects could be related to their capacity to modify DNA methylation patterns.

### Physical Activity

Some studies have investigated the impact of physical activity on DNA methylation status of SM tissue (Table 2). Focusing on genes involved in metabolism and insulin resistance, two studies have reported results from gene-targeted analysis (Alibegovic et al., 2010; Lane et al., 2015). In a cohort of 20 healthy young men, a period of 9 days of bed rest was associated with a general increase in DNA methylation of the promoter region of *PPARGC1A* (peroxisome proliferator-activated receptor-g coactivator-1a) gene, with a tendency toward reversibility after 4 weeks of retraining (Alibegovic et al., 2010). Lane et al. (2015) analyzed the methylation of the gene promoters of *COX4I1* (Cytochrome C Oxidase Subunit 4I1) and *FABP3* (fatty acid-binding protein 3) and observed that an acute exercise of 2 h of cycling was associated with an increase in the methylation of the gene promoters assessed 4 h later. While these two studies focused on genes associated with metabolic pathways in a gene-targeted approach, additional studies have evaluated the effects of physical activity on SM DNA methylation on a wider level. Thus, Barrès et al. (2012) evaluated the effects of a single, acute bout of exercise [completion of a peak pulmonary oxygen uptake rate (VO_2_ peak) test] in biopsies of vastus lateralis SM obtained from 14 healthy, young sedentary individuals. They observed a global decrease in methylation after acute exercise, associated with a marked hypomethylation in promoters of genes known to exert different metabolic and structural functions in skeletal muscle [*PGC-1a* (Peroxisome proliferator-activated receptor gamma coactivator 1 a), *TFAM* (Transcription Factor A, Mitochondrial), *PDK4* (Pyruvate Dehydrogenase Kinase 4), and *PPAR-d* (Peroxisome proliferator-activated receptor d)], in line with the previous results (Alibegovic et al., 2010). The alteration in methylation with exercise was dose-dependent [acute exercise trials at 40% (low-intensity) or 80% (high-intensity) VO_2_ peak] and was associated with higher expression levels. One possible explanation for the decrease in methylation levels after intense acute exercise could be related to the processes of damage and repair: exercise can induce SM damage (Clarkson and Hubal, 2002) and it was previously observed that the mechanisms of repair established after DNA damage could modify the DNA methylation patterns (Russo et al., 2016). However, to our knowledge, this hypothesis has never been directly tested in SM exercise-induced damage.

**TABLE 2:** DNA methylation profiles of skeletal muscle in relation to physical activity.

Author	Year	Number of subjects	Age (years)	Females, *n* (%)	Location		Population	Physical intervention	Tissue for DNA methylation analysis	DNA methylation analysis	Main result
**Candidate gene analysis**											
Alibegovic et al.	2010	20	Mean = 25.0 (SD = 1)	0 (0%)	Denmark	Healthy subjects		9 days of bed rest followed by 4 weeks of retraining	Skeletal muscle	Bisulfite sequencing	Increase in DNA methylation of the promoter region of *PPARGC1A* after bed rest, partially reversed by 4 weeks of retraining
Lane et al.	2015	7	NA	0 (0%)	Sweden	Competitive endurance-trained cyclists		120 min steady-state ride	Skeletal muscle	Bisulfite sequencing	Acute exercise of 2 h of cycling associated with an increase in the methylation of the gene promoters *COX4I1* and *FABP3*
**Genome-wide analysis**											
Nitert et al.	2012	28	Mean = 37.5	0 (0%)	Sweden	Healthy subjects		6 months of exercise	Skeletal muscle	MeDIP-Chip analysis	Methylation levels of 134 genes in SM changed by 6 months of supervised moderate aerobic exercise
Barrès et al.	2012	14	Mean age = 25 (SD = 1)	NA	Sweden	Healthy subjects		Acute exercise	Skeletal muscle	LUMA (luminometric methylation assay) and methylated DNA Immuno- precitation (MeDIP) followed by quantitative PCR	Global methylation decreased after acute exercise. Modification of promoter methylation of exercise-responsive genes in a dose-dependent manner
Rowlands et al.	2014	17	Mean age = 49 (SD = 5)	13 (76.5%)	Polynesia	Type 2 diabetes obese patients		Resistance training or endurance training for 16 weeks	Skeletal muscle	Illumina Methylation 450K	Global decrease in methylation levels with chronic endurance training. Specific modifications in genes involved in lipid and glucose processing pathways
Lindholm et al.	2014	23	Mean = 27.0 (SD = 0.79)	11 (47.8%)	Sweden	Healthy subjects		3 months training (only one leg)	Skeletal muscle	Illumina Methylation 450K	Endurance training reshaped the epigenome. 4919 CpG sites differentially methylated in the trained leg
Seaborne et al.	2018	8	Mean = 27.6 (SEM = 2.4)	0 (0%)	United Kingdom	Healthy subjects		Acute bout of resistance exercise, followed by 7 weeks of resistance exercise, 7 weeks of exercise cessation and a further period of 7 weeks resistance exercise	Skeletal muscle	Illumina Methylation EPIC array	Hypomethylation across the genome after training. Increase in the number of epigenetically modified sites after re-loading
Sailani et al.	2019	16	Mean = 62 (range: 60–65)	0 (0%)	Denmark	Healthy subjects		Comparison of subjects who had performed lifelong regular exercise and subjects who remained sedentary	Skeletal muscle	Whole genome bisulfite sequencing	DNA methylation was lower in 714 gene promoters of the physically active men as compared with the inactive ones

*SD, standard deviation; SEM, standard error of the mean; SM, skeletal muscle; NA, not applicable.*

While Barrès et al. (2012) investigated the effects of acute exercise, similar results were reported for multi-session training (Nitert et al., 2012; Lindholm et al., 2014; Rowlands et al., 2014; Seaborne et al., 2018). Nitert et al. (2012) observed that 6 months of supervised moderate aerobic exercise (3 h per week) changed the methylation levels of 134 genes in SM of men with or without familial history of type 2 diabetes. Most of the genes differentially methylated after exercise (115 out of 134) showed a pattern of decrease in methylation levels. These genes were mostly involved in retinol metabolism, calcium-signaling pathway, and starch and sucrose metabolism (Nitert et al., 2012). In another study, chronic endurance training was also associated with a global decrease in methylation levels and with specific epigenetic modifications in genes involved in lipid and glucose processing pathways (Rowlands et al., 2014). More recently, Lindholm et al. (2014) performed an EWAS in a well-controlled human interventional study on 23 healthy young volunteers, evaluating the impact of a long-term (3 months) endurance exercise training (45 min, four sessions per week). To limit potential confounding factors, authors used an innovative approach and obtained an intra-individual control, training only one leg per subject. Endurance training reshaped the epigenome and 4919 CpG sites across the genome were differentially methylated in the trained leg (Lindholm et al., 2014). Ontology analysis revealed an enrichment in genes associated with structural remodeling of the muscle and glucose metabolism (CpG sites with increased methylation), and with inflammatory and immunological processes or transcriptional regulation (CpG sites with decreased methylation) (Lindholm et al., 2014). Recently, it has also been suggested that lifelong physical activity was able to induce hypomethylation in promoters of genes involved in energy metabolism, myogenesis or oxidative stress resistance (Sailani et al., 2019). Finally, in agreement with the previously mentioned studies which observed a decrease in methylation levels after exercise, Seaborne et al. (2018) identified also hypomethylation across the genome after training. Interestingly, authors measured DNA methylation levels at four different stages in the same eight previously untrained male participants: at baseline and after 7 weeks of resistance training (loading), but also after cessation of resistance exercise (unloading) and return to 7 weeks of resistance training (reloading). Resistance exercise at high intensity induced muscle hypertrophy and significantly modified the methylation levels of 17 365 CpG sites. Importantly, changes in DNA methylation patterns (and specially hypomethylation) were retained even when exercise ceased and following reloading, authors observed an increase in the number of epigenetically modified sites (27 155 CpG sites) and an enhanced number of hypomethylated ones. Combining the epigenomic data with transcriptomic ones, the authors recently observed a significant overlap between genes harboring differential methylation levels after acute or chronic exercise and genes differentially expressed in these conditions (Turner et al., 2019). They identified particularly five genes (*FLNB, MYH9, SRGAP1, SRGN*, and *ZMIZ1*) with persistent hypomethylation after exercise associated with an increased gene expression. These two recent studies show that exercise-induced epigenetic changes can be retained, and that DNA methylation could underpin the capacity of SM to retain information into later life and to respond differently to previously encountered stimuli. This concept was referred to as SM “epi”-memory (Sharples et al., 2016).

### Nutrition

To our knowledge, only one study, focused on the contribution to the development of metabolic diseases, has evaluated the effect of diet on genome-wide DNA methylation patterns in human SM (Jacobsen et al., 2012). Participants of this study were 21 healthy young men, subjected to a short-term high-fat overfeeding diet (HFO) (50% extra calories distributed as 60% fat, 32.5% carbohydrate, and 7.5% protein). Muscle biopsies were obtained after the intervention (5 days of HFO) and after a control diet, in a randomized crossover setting, and were analyzed with the Illumina^®^ Infinium Bead Array 27K. It was observed that HFO induced widespread DNA methylation changes in SM, affecting 6 508 genes. These changes were only partly and non-significantly reversed when the HFO was switched back to the control diet for 6–8 weeks.

## Use of Epigenetic Clocks in the Assessment of Physical Functioning and Sarcopenia in Older Individuals

One critical area of research in the field of sarcopenia is the identification of potential biomarkers, especially for the early selection of patients at risk and the personalized evaluation of the effectiveness of prevention and treatment measures (Curcio et al., 2016). Until now, a valid and unique biomarker of sarcopenia has not yet been identified, but molecular mechanisms associated with the aging process could provide effective ones. During the past years, there has been considerable interest in epigenetic biomarkers of aging, also referred to as epigenetic clocks. Epigenetic clocks are mathematical models which combine weighted averages of methylation levels at specific CpG sites, in order to estimate the biological age, also named epigenetic age or DNA methylation (DNAm) age, of an individual (Field et al., 2018). The epigenetic age predicted according to the different models developed so far is strongly associated with chronological age in several independent studies. The discrepancy between DNAm age and chronological age has been proposed as an index of accelerated or decelerated aging. Epigenetic biomarkers have outperformed other molecular biomarkers in predicting age, and are considered as the most promising biomarkers of biological age (Jylhävä et al., 2017). One of the most important characteristic of the Horvath’s and Hannum’s clocks (Horvath, 2013; Hannum et al., 2013), which are the most popular epigenetic age predictors, is their ability to predict all-cause mortality independent of classic risk factors (Marioni et al., 2015a; Chen et al., 2016; Christiansen et al., 2016; Perna et al., 2016). Thus, individuals whose clock measure is 5 years above their chronological age have a 21% increase mortality risk (Marioni et al., 2015a). Compelling evidence has also accumulated that epigenetic age may be a powerful predictor for age-related diseases and so far, many phenotypes, such as obesity (Horvath et al., 2014; Nevalainen et al., 2017), menopause (Levine et al., 2016), or Parkinson disease for example (Horvath and Ritz, 2015), have been linked to the epigenetic age predictors (Horvath and Raj, 2018). Epigenetic clocks could identify subjects who age at faster rates compared to others and who are therefore more at risk to develop adverse effects of aging, such as sarcopenia or frailty. Frailty is defined as a state of increased vulnerability, that results from an age-related decline in reserve and function across multiple physiologic systems (Xue, 2011). Sarcopenia and frailty are both associated with negative health outcomes and sarcopenia is considered as a major risk factor for frailty (Cesari et al., 2014). The biological clocks may shed light on mechanisms behind accelerated decline in physical functioning associated with sarcopenia and frailty and can represent a potential tool able to track individual variation in physical function with aging. Potential relationships between epigenetic age acceleration measures and frailty-related phenotypes have been investigated and the results obtained are summarized in the next paragraphs.

So far, five studies have examined associations between measures of epigenetic age acceleration (AA) in blood and fitness measures of aging (Marioni et al., 2015b; Quach et al., 2017; Simpkin et al., 2017; Gale et al., 2018a; Sillanpää et al., 2018; Table 3). Marioni et al. (2015b) were the first to examine cross-sectional and longitudinal associations between the epigenetic clock (Horvath’s and Hannum’s predictors) with walking speed and HGS. At baseline, a higher epigenetic AA was significantly associated with a weaker grip strength, while the association with walking speed was non-significant. Walking speed and HGS declined moderately over time but epigenetic AA did not correlate with changes over 6 years of follow-up (Marioni et al., 2015b). It should be noted that not all participants had DNA methylation data available in the subsequent waves of analysis after baseline, possibly contributing to limit the statistical power to test the associations between changes in methylation age and changes in fitness. Simpkin et al. (2017) reported different results: they evaluated the associations between epigenetic AA at age 53 and changes in objective measures of physical performance (HGS, standing balance time and chair rise speed) from ages 53 to 60–64. In this study, cross-sectional data revelated no association between physical performance at age 53 and epigenetic AA, while an association between AA and a greater decrease in HGS in British females aged between 53 and 60–64 was noted. For a one-year increase in epigenetic AA, HGS decreased by an additional 0.42 kg. No association was found with standing balance time or chair rise speed (Simpkin et al., 2017). In a cohort of 48 monozygotic twin sisters, Sillanpää et al. (2018) observed that an increased epigenetic AA was also associated with a lower HGS, but not with knee extension strength or walking speed. Lately, refinements in Horvath’s and Hannum’s predictors, termed extrinsic and intrinsic epigenetic AA (EEAA and IEAA, respectively), which take into account variations in the cellular composition (cell counts) when measuring DNA methylation from whole blood, have been developed. IEAA is independent of age-related changes in blood cell composition, while EEAA incorporates them. Quach et al. (2017) have investigated the relationship between these two newly developed estimates of biological age and levels of physical activity (categorized as sedentary or active). They analyzed cross-sectional data from 4 173 postmenopausal female participants from the Women’s Health Initiative and 402 participants from an Italian population. They found a weak correlation between being biologically older (greater EEAA) and being physically inactive (Quach et al., 2017). It should be noted that the data on physical activity levels were self-reported. When sedentary and walking behavior in older people were objectively measured over 7 days using an activPAL activity monitor, no convincing evidence was observed on a possible association between biological age, estimated by IEEA or EEAA, and the amount of time participants spent being sedentary of physically active (Gale et al., 2018a).

**TABLE 3:** Epigenetic biomarkers of aging and markers of physical fitness.

Author	Year	Number of subjects	Age (years)	Females, *n* (%)	Location	Population	Measure of physical functioning	Tissue for DNA methylation analysis	DNA methylation analysis	Main result
**Physical functioning**										
Marioni et al.	2015b	1091	Mean = 69.5 (SD = 0.83)	543 (49.8%)	Scotland	Lothian Birth Cohort 1936 (individuals born in 1936 who were living in the Lothian area of Scotland)	Walking speed. HGS	Whole blood	Illumina Methylation 450K. Horvath’s clock.	*Cross-sectional study*: Higher AA significantly associated with weaker HGS *Longitudinal study*: No correlation between AA and changes over 6 years of follow-up
Simpkin et al.	2017	790	Mean = 53.4 (SD = 0.16)	790 (100%)	United Kingdom	MRC National Survey of Health and Development (NSHD). Individuals born in the same week of March 1946	HGS. Standing balance time. Chair rise time	Buccal cell (*n* = 790) and matched blood tissue (*n* = 152)	Illumina Methylation 450K. Horvath’s clock.	*Cross-sectional study*: No association between physical capability at age 53 and epigenetic AA *Longitudinal study*: For a 1-year increase in AA, decrease in HGS by an additional 0.42 kg from age 53 to 60–64
Quach et al.	2017	*WHI sample*: 4173. *InCHIANTI study*: 402	*WHI study*: Mean = 64 (SD = 7.1) *InCHIANTI study*: Mean = 71 (SD = 16)	*WHI study*: 4173 (100%). *InCHIANTI study*: 229 (56%)	*WHI study*: United States. *InCHIANTI study*: Italy.	Post-menopausal women	Self-reported physical activity	Whole blood	Illumina Methylation 450K. Horvath’s and Hannum’s clocks. IEAA. EEAA.	Weak correlation between greater EEAA and being physically inactive
Sillanpää et al.	2018	48	Mean = 61.3 (SD = 5.9)	48 (100%)	Finland	Monozygotic Caucasian twin pairs. Participants originate from two studies [SAWEs (*n* = 15 pairs) and FITSA (*n* = 9 pairs)]	HGS. Knee extension strength. 10 m maximal walking speed test	White blood cells	Illumina Methylation 450K. Horvath’s clock.	Increased epigenetic AA associated with lower HGS
Gale et al.	2018a	248	Mean = 79.0 (SD = 0.45)	122 (47.1%)	Scotland	Lothian Birth Cohort 1936 (individuals born in 1936 who were living in the Lothian area of Scotland)	Percent of daily time spent sedentary. Number of sit-to-stand transitions	Whole blood	Illumina Methylation 450K. Horvath’s and Hannum’s clocks. IEAA. EEAA.	EEAA or IEAA not associated with objectively measured sedentary or walking behavior
**Frailty**										
Breitling et al.	2016	1820	*Dataset 1*: Mean = 62.1 (SD = 6.5) *Dataset 2*: Mean = 63.0 (SD = 6.7)	*Dataset 1*: 484 (50.0%). *Dataset 2*: 464 (54.5%)	Germany	ESTHER study: observational study of the elderly general population of Saarland	Frailty Index based on the accumulation of deficits	Whole blood	Illumina Methylation 450K. Horvath’s clock.	Greater epigenetic AA associated with frailty For a one-year increase in epigenetic AA, frailty index increased by about 0.25%
Gale et al.	2018b	791	Mean = 69.5 (SD = 0.84)	398 (50.3%)	Scotland	Lothian Birth Cohort 1936 (individuals born in 1936 who were living in the Lothian area of Scotland)	Fried frailty criteria	Whole blood	Illumina Methylation 450K. Horvath’s and Hannum’s clocks. IEAA. EEAA.	Greater EEAA associated with an increased risk of being frail. For a one-year increase in EEAA, RR for being frail compared to being not frail = 1.06 (1.02 − 1.10)

*SD, standard deviation; HGS, hand grip strength; AA, age acceleration; IEAA, intrinsic epigenetic age acceleration; EEAA, extrinsic epigenetic age acceleration.*

Two studies have specifically evaluated the relationship between the epigenetic AA in blood and frailty (Table 3) and their results were globally consistent (Breitling et al., 2016; Gale et al., 2018b). In a cohort of 1 820 older adults, Breitling et al. (2016) observed that a greater epigenetic AA was associated with frailty, measured by a deficit accumulation-based approach, even after accounting for several risk factors and blood cell counts. For a one-year increase in epigenetic AA, the frailty index increased by about 0.25%. Two years later, Gale et al. (2018b) reported the results from the Lothian Birth Cohort 1936. They observed that having a greater EEAA was associated with an increased risk of being frail, as defined by Fried criteria (Fried et al., 2001). For a one-year increase in EEAA, the risk ratio for being frail compared to being not frail was 1.06 (CI 1.02–1.10). No associations were found with pre-frail status, or with IEEA measures (Gale et al., 2018b).

Interestingly, there is an overlap between some CpG sites included in the epigenetic signature of muscle aging published by Zykovich et al. (2014) and some CpG sites included in Hannum’s clock (Hannum et al., 2013). Over the 500 CpG sites able to distinguish SM samples from old or young subjects, nine are also present in the epigenetic age predictor. Among them, three CpG sites related to *FHL2* (cg22454769, cg24079702) and *ELOVL2* (cg16867657) genes were previously identified as strongly correlated with age in several tissues (Garagnani et al., 2012; Bacalini et al., 2017; Spólnicka et al., 2018), while two other ones (cg10501210 and cg07553761 associated with genes *C1orf132* and *TRIM59*, respectively) were also previously integrated in a forensic age predictor (Spólnicka et al., 2018). These CpG sites have a high age prediction accuracy and are poorly affected by an eventual disease status (Spólnicka et al., 2018).

## Limitations and Future Directions

**FIGURE 1:**
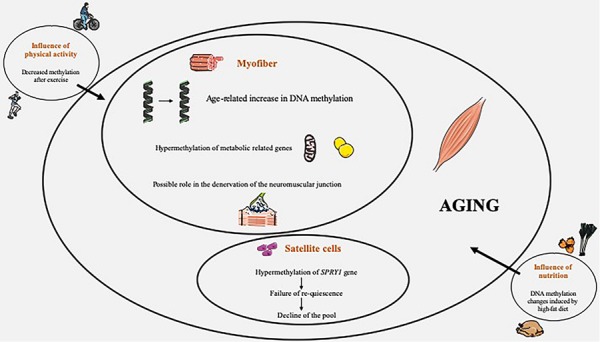
Overview of DNA methylation changes occuring in skeletal muscle during aging, with influence of physical activity and nutrition.

We presented here a focused review of the available experimental evidences linking DNA methylation and SM aging in humans, as well as the impact of exercise, acute or long-term, and diet (Figure 1). It is evident that current knowledge in this field is still sparse and that future research is imperative to further better elucidate the connection between DNA methylation remodeling and SM aging in humans. We believe that there is a need to pursue research in this field. In the past years, a wealth of studies, based notably on technological innovations such as microarrays or high-throughput sequencing, has clearly demonstrated a powerful link between complex epigenetic changes and aging, and epigenetic alterations are now doubtless acknowledged as a crucial hallmark of aging (López-Otín et al., 2013; Kennedy et al., 2014). Moreover, epigenetics, modulated by external factors, are now recognized as a fundamental link between environment and aging, and DNA methylation is an appealing target for therapeutic interventions. Given this particular importance, it is clearly pertinent to better decipher the DNA methylation changes occurring with age in SM.

Moreover, the characterization of age-related DNA methylation changes has allowed, in the past years, the identification of epigenetic markers of biological age (Hannum et al., 2013; Horvath, 2013; Levine et al., 2018), which are currently the most promising biomarkers of aging and life expectancy and may increase our understanding on human aging (Horvath and Raj, 2018). Literature data presented in the above sections provide some evidence suggesting that epigenetic AA could be a marker of physical performance in older individuals, associated specially with frailty and HGS. HGS has already proven its utility in clinical practice and has been associated with mortality (Cooper et al., 2010; Ling et al., 2010). The concomitant assessment of epigenetic age, in a minimally invasive manner in routinely obtained blood, could complement the clinical assessment of physical performance and being the starting point of a “biological geriatric assessment” (Tuttle and Maier, 2018), in order to track individual variation in physical function with aging.

It is sure that our knowledge in the field of DNA methylation and SM aging is still limited and that much has to be done, in particular to overcome some important limitations. One of the main limitations of this topic is that the available studies are mostly descriptive, considering only directional changes of DNA methylation and lack mechanistic insights. In this context, it is hard to conclude to which extent the observed DNA methylation changes in SM are a cause or a consequence of the aging process. The age-related changes in epigenetic patterns could contribute to aging by affecting genomic stability and gene expression regulation. The contribution of the age-related changes in DNA methylation to transcriptomic changes is highly dependent on the genomic context, as promoter methylation is usually associated with gene silencing while DNA methylation changes occurring at other sites have a more variable impact on gene transcription (Jones, 2012). Regarding the influence of physical activity and diet, the available studies were mainly focused on the possible influence between epigenetic modifications, lifestyle factors and genes involved in metabolic adaptation and were not specifically designed to evaluate the aging process. Except for the study recently published by Sailani et al. (2019), they were all performed in young, healthy, disease-free subjects and to date, to our knowledge, no interventional study has examined the effects of physical activity or nutrition on DNA methylation profiles of SM in elderly, or link the observed epigenetic changes to phenotypic changes and health-related outcomes following training.

Additionally, just as other studies that have analyzed the association between epigenetic changes and age-related phenotypes, some of the DNA methylation studies mentioned in the previous sections on muscle aging and physical functioning have been carried out on whole blood samples, comprised of multiple cell types. Cell composition of the analyzed sample is a well-known source of heterogeneity in DNA methylation studies (Ziller et al., 2013) and some age-related DNA methylation changes are tissue-specific (Slieker et al., 2018). Whole blood is the most accessible source of samples in humans and the invasive surgical procedure required to obtain SM is frequently considered as a limiting factor for the study of this tissue in humans. However, it should be kept in mind that the use of whole blood could represent a limit to infer mechanistic insights from the data obtained and renders a direct comparison of these studies more difficult.

Differences between gender balance of the subjects included could add another layer of difficulty to compare between the different studies. It was previously reported substantial differences in gene expression or protein-protein interactions in human SM according to gender during aging (Roth et al., 2002; Welle et al., 2008; Liu et al., 2010, 2013; Shafiee et al., 2018), and a recently published study also observed for the first time differences in DNA methylation between myoblasts and myotubes of males and females (Davegårdh et al., 2019).

Regarding epigenetic biomarkers of aging, some limitations exist too, and they should be overcome before epigenetic clocks can be used routinely for physical functioning and sarcopenia. Firstly, the size of the observed associations is small, and the cross-sectional nature of the majority of the published studies does not allow to draw firm conclusions on predictive properties of the clocks on sarcopenia or frailty incidence. Moreover, the actual high cost of these markers seems to be poorly adapted to clinical application.

The limited number of studies available highlights how young this field is, but an increasing number of papers are being published really recently (Sailani et al., 2019; Turner et al., 2019) and the future holds exciting promises. Further investigation is warranted and should address the above-mentioned limitations. The functional impact of the age-related methylation changes in SM must be studied. Moreover, DNA methylation patterns of sarcopenic SM should be evaluated. Indeed, individuals included in the studies published so far are predominantly healthy and disease-free, and it would be of great interest to compare the DNA methylomes of sarcopenic and non-sarcopenic SM in older adults, in order to identify targets that could differ from healthy muscle aging. Additionally, the tissue, or even maybe the fiber, specificity of the age-associated DNA methylation changes deserves deeper investigation.

Regarding the impact of physical activity and nutrition, future studies are required to evaluate if training or diet are able to modify DNA methylation in the specific population of elderly and if these lifestyle factors are able to reverse the age-related DNA methylation signatures reported during muscle aging. It remains also to be evaluated the retainability/reversibility rates of these processes and to what extend the exercise- or diet-induced methylation changes are involved in the beneficial effects of training and diet.

Finally, regarding the epigenetic biomarkers of aging, large powered longitudinal human studies, with several measurements at different time points, are required. Many developments of the epigenetic clocks are anticipated in the coming years, as new epigenetic biomarkers of aging are constantly emerging and could be interesting tools for physical functioning. For example, a new interesting epigenetic biomarker, termed DNAm PhenoAge, was recently published with the intention to better characterize lifespan and healthspan (Levine et al., 2018). This model, based on the replacement of chronological age with a “phenotypic age” constructed with a weighted average of 10 clinical characteristics, outperformed the previous versions of the clocks in terms of prediction of all-cause mortality and age-related morbidity, and appeared related to exercise, dietary habits and physical performance (Levine et al., 2018). This model appears to be an extremely interesting target to evaluate in the years to come.

## Conclusion

Epigenetic changes have an important influence on the aging process and they represent one crucial hallmark of aging (López-Otín et al., 2013; Kennedy et al., 2014). Our current knowledge on how age-associated DNA methylation changes are related to muscle aging is still sparse. Further research is needed to disentangle the role of epigenetics in muscle aging, and to investigate how skeletal muscle methylome can change in response to exercise or dietary interventions. A proper understanding of the pathways involved in muscle aging and sarcopenia is required to pave the way for the development of new strategies for diagnosis and treatment of the deleterious effects of muscle loss with age.

## Author Contributions

All authors conceived the study, analyzed the literature, and discussed and wrote the manuscript.

## Conflict of Interest Statement

The authors declare that the research was conducted in the absence of any commercial or financial relationships that could be construed as a potential conflict of interest.
